# Study of a QCM Dimethyl Methylphosphonate Sensor Based on a ZnO-Modified Nanowire-Structured Manganese Dioxide Film

**DOI:** 10.3390/s100908275

**Published:** 2010-09-02

**Authors:** Zhifu Pei, Xingfa Ma, Pengfei Ding, Wuming Zhang, Zhiyuan Luo, Guang Li

**Affiliations:** 1 National Key Laboratory of Industrial Control Technology, Department of Control Science and Engineering, Zhejiang University, Hangzhou 310027, China; E-Mails: florapzf@yahoo.com.cn(Z.P); dpfzjuv@126.com(P.D.); guangli@zju.edu.cn (G.L.); 2 State Key Laboratory of Silicon Materials, Zhejiang University, Hangzhou 310027, China; E-Mail: xingfamazju@yahoo.com.cn (X.M.); 3 School of Environmental and Material Engineering, Center of Advanced Functional Materials, Yantai University, Yantai 264005, China; 4 Computer Learning Research Centre, Royal Holloway, University of London, Egham, Surrey TW20 0EX, UK; E-Mail: Zhiyuan.Luo@cs.rhul.ac.uk (Z.L.)

**Keywords:** quartz crystal microbalance, gas sensor, volatile organic vapor, DMMP, nanowire, manganese dioxide, zinc oxide

## Abstract

Sensitive, selective and fast detection of chemical warfare agents is necessary for anti-terrorism purposes. In our search for functional materials sensitive to dimethyl methylphosphonate (DMMP), a simulant of sarin and other toxic organophosphorus compounds, we found that zinc oxide (ZnO) modification potentially enhances the absorption of DMMP on a manganese dioxide (MnO_2_) surface. The adsorption behavior of DMMP was evaluated through the detection of tiny organophosphonate compounds with quartz crystal microbalance (QCM) sensors coated with ZnO-modified MnO_2_ nanofibers and pure MnO_2_ nanofibers. Experimental results indicated that the QCM sensor coated with ZnO-modified nanostructured MnO_2_ film exhibited much higher sensitivity and better selectivity in comparison with the one coated with pure MnO_2_ nanofiber film. Therefore, the DMMP sensor developed with this composite nanostructured material should possess excellent selectivity and reasonable sensitivity towards the tiny gaseous DMMP species.

## 1. Introduction

Sensitive and selective detection of a wide variety of chemical species has become a necessity in many applications, including the quantification of chemical warfare agents (CWAs), explosives, environmental pollutants and many other toxic industrial compounds [1,2]. The threat of terrorism has greatly increased the need for fast detection of CWAs, so it is urgent to develop CWA sensors with fast response, high specificity, low detection limits and easy operation [3,4]. Dimethyl methylphosphonate [DMMP, CH_3_PO(OCH_3_)_2_] due to its nontoxicity and organophosphorus compound elemental composition that mimics nerve agents, is commonly considered as a simulant for CWAs and insecticides, such as the G-series nerve agents tabun (GA), sarin (GB), soman (GD) and paraoxon [5]. DMMP has also become a significant environmental and food chain pollutant due to its large consumption as a common additive for anti-foaming agents, plasticizers, stabilizers, textile conditioners and antistatic agents [2]. Consequently, DMMP sensors with high sensitivity, rapid response, low energy consumption and good reversibility at room temperature are highly desirable, not only for neurotoxin detection for counter-terrorism purposes, but also for environmental protection and medical diagnoses for risk management [6–8].

Focusing on highly sensitive functional materials with advanced fabrication technology, metal oxide semiconductors [8–11], carbon nanotubes [12–14], conducting polymers [15] and organometallic compounds [16,17] have been studied. Of these, metal oxides are well-known for their industrial applications as adsorbents, catalysts and catalyst supports, especially manganese dioxide (MnO_2_) and titanium dioxide (TiO_2_) which are widely used as molecular sieves and electrode materials in batteries and sensors due to their unique electronic and surface properties [18–22]. Furthermore, they are often used as sensing materials for different gases now due to their large gases adsorbent capacity [8,23,24]. Several investigations have been carried out concerning the adsorption and reaction of DMMP or other CWAs on the surfaces of different metal oxides, including MgO [25,26], Al_2_O_3_ [26–28], TiO_2_ [26,29–31], Fe_2_O_3_ [32,33], ZnO [26,34,35], SiO_2_ [36], MnO_2_ [37] and WO_3_ [26,38]. Zinc oxide (ZnO) in particular shows very strong sensitivity toward toxic substances, such as halogens, sulfur, volatile organic compounds [35] and organophosphorus compounds [34,35]. Furthermore, the experiments on powdered TiO_2_ have revealed three distinct modes of adsorption: DMMP condenses on the outer surface of TiO_2_ below 160 K; molecularly diffuses into the TiO_2_ interior and chemisorbs on TiO_2_ from 160 to 200 K; and dissociatively chemisorbs above 214 K [30]. Enhancement to the absorption and reactivity of their nanoparticles and other nanostructures to sensitively detect various pollutants and harmful substances, including organophosphorus compounds, is anticipated due to their unique electronic properties, morphological features and high surface area [39]. These details indicate the nanostructured metal oxides like TiO_2_, ZnO and MnO_2_ may show sensitive and dissociative adsorbent of gas-phase DMMP at ambient temperature, promising sensing behavior for the mass detection of gas-phase organophosphorus compounds. However, only a few experimental studies have been reported on the adsorption of toxic chemicals or chemical warfare agent simulants on nano-structured MnO_2_ at ambient temperature [40].

In addition, it is interesting that the behavior of alumina-supported iron oxide may be significantly different from that of pure alumina [32,33]. Furthermore, zinc oxide doped in SnO_2_ may improve the reliability and sensitivity of the SnO_2_ sensors for simulants of the CWAs at 250 to 400 °C [40]. It is suggested that the absorption capability of DMMP on MnO_2_ surface is possibly improved by ZnO modification. In this study, we attempted to modify the MnO_2_ nanostructure with ZnO to explore new sensing materials and furthermore to evaluate the DMMP absorption properties at ambient temperature by constructing a sensitive DMMP sensor based on the MnO_2_ nanostructured film and the quartz crystal microbalance (QCM). Consequently, the adsorption behavior of DMMP on the composite material was characterized through the detection of tiny organophosphonate compounds with QCM sensors coated with ZnO-modified MnO_2_ nanofibers and the comparison of these properties to those of the same sensors coated with pure MnO_2_ nanofibers. We thus concluded that these features make the developed DMMP sensor possess excellent selectivity and reasonable sensitivity.

## 2. Experimental Section

### 2.1. Materials

Chemicals and regents for nanostructured metal oxides were manganese (II) sulfate (MnSO_4_), potassium permanganate (KMnO_4_), zinc nitrate [Zn(NO_3_)_2_], hexamethylenetetramine and ammonia. These chemicals and reagents were analytical grade and commercially available. The chemicals for volatile organic vapors (VOCs) were dimethyl methylphosphonate (DMMP), acetone, *p*-dichlorobenzene (*p*-DCB), *p*-dimethylbenzene (*p*-xylene), ethanol, *n*-hexane and trichloro-methane (chloroform) purchased from Sigma-Aldrich (Shanghai, China) and Wako Pure Chemicals (Osaka, Japan). All these chemicals and reagents were used directly as received without further purification. The AT-cut 6.0 MHz (HC-49/U) quartz crystals with aluminum electrodes on both sides were purchased from Hosonic International (Hangzhou) Ltd., China. The crystals were rinsed by ethanol and then deionized water prior to use. All experiments were carried out at room temperature (about 25 degrees Celsius in an air-conditioned room).

### 2.2. Preparation of pure and ZnO-modified MnO_2_ NW-structured films

#### 2.2.1. Preparation of NW-structured MnO_2_

In the preparation process, 1.0 g MnSO_4_ and 0.5 g of oxidizing reagent (KMnO_4_) were dissolved in 20 mL of distilled water at room temperature to form a homogeneous solution. The solution was then transferred into a 100 mL Teflon-lined stainless steel autoclave, sealed and maintained at 120 °C for about 24 hours. After the resulting solid product was filtered and washed with distilled water to remove the possibly remnant ions in the final products and finally dried in air, MnO_2_ NWs, the final product was obtained.

#### 2.2.2. Modification of NW-structured MnO_2_ with ZnO

About 0.5 g of home-made MnO_2_ NWs was dispersed in 20 mL distilled water; sequentially, 0.5 g zinc nitrate and 0.5 g hexamethylenetetramine were added. The mixture was then transferred into a Teflon-lined stainless steel autoclave. The pH value of reaction solution was adjusted to around 10 with ammonia. The hydrothermal treatments were carried out at 90–95 °C for 5 hours. After the resulting solid product was filtered, washed with distilled water repeatedly, and finally dried in air, the ZnO-modified MnO_2_ nanowires (NWs) were obtained.

#### 2.2.3. Preparation of QCM sensors with ZnO-modified MnO_2_ NW-structured film

About 1 mg of ZnO-modified NW-structured MnO_2_ was weighed and dispersed in deionized water to form a dark brown colored stock solution with a concentration of 2 μg/μL. After standing for 24 hours, 2.5, 5, 7.5, 10, 12.5 or 15 microliters of the aqueous solution was dispensed onto the electrode surface of QCMs using a micropipette, forming a sensing film with an area of 0.2 cm^2^ and a thickness index of 25, 50, 75, 100, 125 or 150 μg/cm^2^, respectively. Then the device was dried in a dry cabinet at room temperature. After these steps, the QCM sensors coated with ZnO-modified NW-structured MnO_2_ film were obtained.

#### 2.2.4. Structural characterization of the pure and ZnO-modified MnO_2_ films on QCMs

The nanostructure of MnO_2_ films were characterized by scanning electron microscopy (SEM). This observation was performed using a Field-Emission Scanning Electron Microscope with Energy Dispersive Spectrometer (FESEM-EDS, HITACHI S4800, Japan), operated at 25.0 kV. Both the pure and ZnO-modified MnO_2_ films on the QCM sensors were deposited by platinum on the surface for SEM observation. The NW-structures of MnO_2_ films were observed and recorded.

### 2.3. Experimental procedure for gas sensing

The Sauerbrey equation was developed for oscillation in air and only applies to rigid mass attached to the crystal [41]. It gives the change in the oscillation frequency of piezoelectric quartz (Δ*f*) as a function of the mass (Δm) added to the crystal:

(1) $$\Delta f=-\frac{2f_{0}^{2}}{A\rho_{q}\upsilon_{q}}\Delta m$$

Here, Δ*f* is the observed frequency change (Hz), *f*_0_ is the fundamental resonant frequency of crystal, A is the active area, the area where the crystal is coated with electrodes on both sides, ρ_q_ is the density of quartz and υ_q_ is the shear wave velocity in the quartz. A home-made experimental system was set up to evaluate the as fabricated QCM sensors. The gas sensors set in the 500 mL sealed chamber of experimental setup were thus characterized at around 25 °C with either analytic gases for measurements or high-purity nitrogen gas for cleaning. The sensing-film-coated QCM was used as sensing unit while an uncoated QCM was used in the experimental system as reference. The variation of the frequency difference between the reference and sensing QCMs was defined as the response of QCM gas sensors. When the QCM DMMP sensor was exposed to the analyte DMMP, the sensing film would absorb the analytic gas, therein inducing a decrease in the working resonant frequency of the QCM DMMP sensors and the frequency change increase from a value at the start of the experiment. The working frequency of the QCM DMMP sensor in experimental setup changed from the fundamental resonant frequency of crystal coated with sensing film to a lower steady frequency of crystal decided by the sensing film-absorbed target gas. In this way, as the adsorption process approached equilibrium between the adsorption/desorption at a given concentration of DMMP, the frequency change or the response of QCM sensor increased, and finally reached a plateau phase. According to Equation (1) and adsorption mechanism [30], the steady value of frequency change at the plateau phase would determine the amount of gas absorbed in the film at ambient temperature. The absorbed DMMP analyte could be desorbed by high-purity N_2_, due to its dissociative adsorption at ambient temperature. The sensing QCM would thus be recovered; consequently the frequency change of the sensing QCM would be zeroed.

DMMP, acetone, *p*-DCB, *p*-xylene, ethanol, *n*-hexane and chloroform vapors were used as the analytical gases for this investigation. The experiments were performed as follows: first, a target analyte was injected into the testing chamber, the sensing film then absorbed the analyte, thus decreasing the output frequency of the sensing QCM. The response of the QCM gas sensor, or an increase of the frequency difference between the sensing and reference QCMs to the analyte, was measured continuously at a 1-second interval [42,43]. After the response reached a plateau phase, the measurement course in a cycle was finished. The chamber was then purged with high-purity nitrogen gas to expel the analyte and recover the sensing films of sensors; the cleaning course for next cycle of measurement was started. This process was repeated several times for each analyte to get reliable results.

## 3. Results and Discussion

### 3.1. Morphology of the sensing films

**Figure 1.**
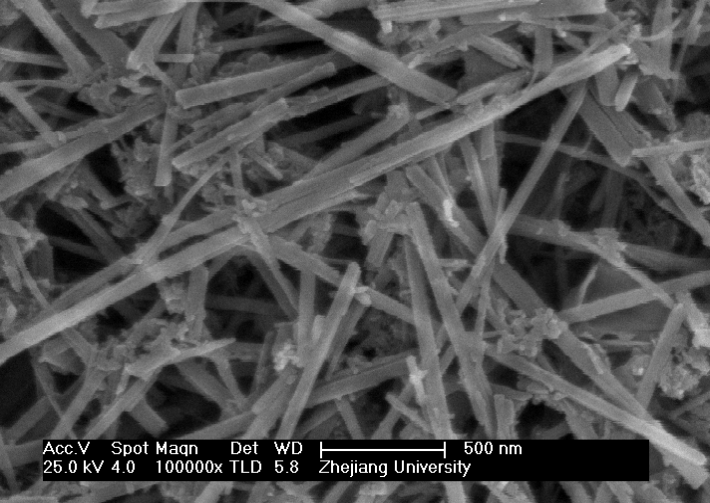
SEM image of the ZnO-modified MnO_2_ nanowire.

Both the pure and ZnO-modified MnO_2_ films on QCM sensors were investigated. The composite nanostructures in the ZnO-modified MnO_2_ film can be seen in Figure 1.

**Figure 2.**
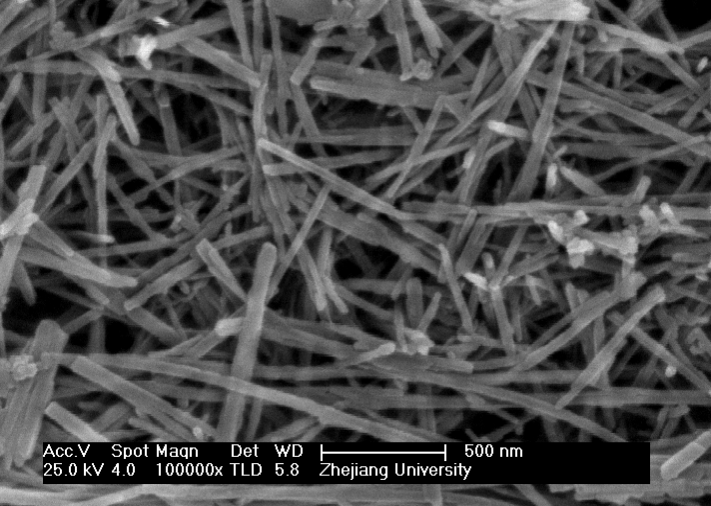
SEM image of the pure MnO_2_ nanowires.

For comparison, the morphology and structure of the pure NW-structured MnO_2_ film is shown in Figure 2. The observed data indicate that the ZnO-nanoparticles modified the surface of MnO_2_ NWs. These SEM images confirmed that the composite nanostructures were formed in the ZnO-modified MnO_2_ film by the ZnO nanoparticles’ joining to the MnO_2_ NWs. Therefore, we attribute the distinct sensing properties of the ZnO-modified MnO_2_ NW-structured QCM sensor to the composite nanostructures of the ZnO nanoparticle-modified surface of the MnO_2_ NWs.

### 3.2. Sensitivity and repeatability of the QCM DMMP sensors

**Figure 3.**
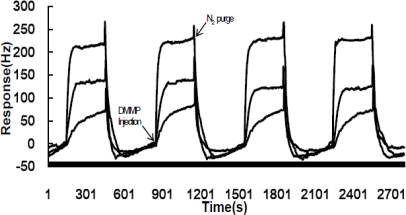
Response cycles of a QCM sensor coated with ZnO-modified NW-structured MnO_2_ film towards 0.35, 0.70 and 1.75 ppms DMMP purged by high-purity nitrogen gas at room temperature.

The QCM sensors based on ZnO-modified NW-structured MnO_2_ films were repeatedly tested at predefined DMMP concentrations for assessing their sensitivity and repeatability. At first, the QCM sensors coated with ZnO-modified nanostructured MnO_2_ films were exposed to a series of defined concentrations of DMMP vapors diluted in high-purity nitrogen gas to assess their sensitivity. This investigation was performed by alternatively exposing the sensors to 300-second DMMP vapors and 400-second nitrogen gas. The responses to the alternating inputs between the DMMP vapors of 0.35, 0.70 or 1.75 ppm and cleaning gas of high purity nitrogen gas at room temperature were recorded and displayed in Figure 3.

The response curves in Figure 3 indicate that the sensors were sensitive to the vapor concentrations and quickly responded to the high-purity nitrogen gas purge, thus possibly possessing a high sensitivity and good repeatability towards gaseous DMMP.

### 3.3. Relationship between the responses (sensitivity) and the thickness of ZnO-modified MnO_2_ NW-structured films

The Sauerbrey equation generally gives a good prediction of the linear responses of the QCMs working in air, but only applies to rigid films [41]. The real situation of our developed QCM gas sensors to DMMP vapor sorption is much more complex, so there might be many challenges in predicting the linear relationship between the QCM sensor responses and the concentrations of DMMP vapors. Clearly, besides the nature and thickness of crystals (such as fundamental resonant frequency and Q value), various factors related to the sensing films and target gases, such as the analyte-surface interaction and thickness of sensing films, can possibly contribute to the sensitivity and linearity of the sensor responses. For the QCM DMMP sensor based on NW-structured MnO_2_ film, the film thickness is an important factor for suitable response when the material nature and fabrication method are given. The thickness of the sensing films on electrodes thus effects on both the mass-sensing properties of QCMs and physicochemical adsorption of DMMP.

**Figure 4.**
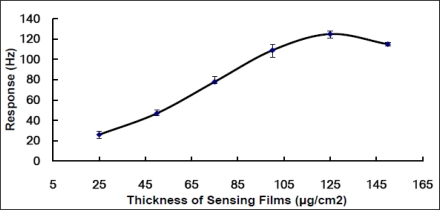
Responses of the QCM sensors with various thicknesses of sensing films to a predefined concentration of DMMP (0.7 ppm).

To address this problem, we designed test experiments to investigate the relationship between the sensitivity of the sensors and the thickness of the sensing films on the sensors. Through these experiments, we could maximize the sensitivity and optimize the linearity of the QCM sensor by appropriately selecting the thickness of sensing films. The electrode surface on crystals was deposited with 2.5, 5, 7.5, 10, 12.5 or 15 microliters of the 2 μg/μL aqueous solution of the ZnO-modified MnO_2_ on 0.2 cm^2^ working area forming sensing films having six different thicknesses. Although the abstract values of these thicknesses could be not figured out due to lack of the mass density of the film, the thickness indices could be easily obtained by supposing that the mass density of film is constant for all films produced with same material and fabrication method. Thus, the ZnO-modified MnO_2_ composite film deposited on sensors had a thickness index of 25, 50, 75, 100, 125 or 150 μg/cm^2^, respectively. This thickness index reflects the variation of film thickness, thus defined and used as thickness in this study. The test experiments were performed during the produced QCM sensor was exposed in 0.7 ppm, a predefined concentration, of gaseous DMMP. The experimental findings, displayed in Figure 4, reveal a significant nonlinear relationship between the sensitivity of the sensors and the thickness of the sensing films.

Here, we tried to explain the nonlinear properties of the QCM sensor through the behaviors of QCM oscillation and DMMP adsorption. Both of them are closely related to the thickness of sensing film deposed on QCM substrate. The nonlinear phenomena seems partly contributed to the interference of the sensing film with the vibration state of QCM substrate. Nevertheless, the Sauerbrey equation still works on the QCM gas sensor. In principle, the responses of QCM sensor generally arise from both gravimetric and viscoelastic changes in real sensing films, whereas the Sauerbrey equation predicts the outputs according to the gravimetric changes in ideal rigid films.

The sensing film moves synchronously with the underlying QCM substrate if the thickness of the coated sensing film is small enough relative to the acoustic wavelength in the film [45]. In this situation, the motion imparted by the QCM substrate displaces the sensing film parallel to the surface of the substrate just as the situation in a rigid film. The responses to the sensing film thus obey the Sauerbrey equation and reflect the gravimetric changes of the sensing film regardless of its shear modulus [46]. On the other hand, when the sensing thickness is big enough or acoustically thick (thicker than a few percents of the acoustic wavelength) the thickness stress gradients will become important. In this situation, the responses to the NW-structured MnO_2_ sensing film will depend on both the acoustic thickness and the thickness shear modulus of the sensing film prior to and during DMMP vapor exposure [45–48], thus no longer accurately obeying the Sauerbrey equation. The experimental results, shown in Figure 4, reveal some of these effects. As is known, these nonlinear phenomena contribute to the variation of the QCM sensor’s static working status consisting of the resonant frequency, Q value and other parameters prior to DMMP vapor exposure, as well as the dynamic sensing status of the film during DMMP vapor exposure, which will be described in the next paragraph. Despite working in a static working point different from that of theoretical crystal, the real QCM DMMP sensor can detect the mass changes of sensing film according to the Sauerbrey equation. That is why the QCM had been utilized in chemical and biological sensors in so many applications for so many years.

In our opinion, the nonlinear phenomena seem to contribute more significantly to the gas-surface interaction and adsorption of DMMP inside the ZnO-modified NW-structured MnO_2_ sensing film. The uniform and porosity structures of the NWs in film lead to a huge surface-to-volume ratios; these ratios, in turn, make the interaction between the sensing film and DMMP vapor efficient, thus, leading to quick and thorough equilibrium analyte absorption/desorption state. When the sensing film on the QCM substrate is thin, the whole sensing film, as an effective surface adsorption area, effectively adsorbs the target analyte. Thus, while the QCM sensor’s responses reflect the gas diffusion and adsorption/desorption process in sensing film exposed in a fixed concentration of DMMP vapor, the QCM sensor’s plateau responses reflect equilibrium status of adsorption/desorption to a given DMMP concentration. The readouts of the plateau curve are consequently the responses of the QCM sensor to a given DMMP concentration. The effective surface adsorption area will increase together with the amount, or the thickness of sensing materials deposited on a given area of substrate. Therein, the total amount of the DMMP absorbed, furthermore, the responses of the sensor exposed in a given DMMP concentration will also increase together with the thickness of sensing materials, as the linear line between 0.03 and 125 μg/cm^2^ shown in Figure 4. When the thickness is big, the DMMP molecule will penetrate a larger depth inside the sensing film to diffuse to the bottom of sensing film and reach equilibrium status of adsorption/desorption in whole sensing film. The responses of QCM sensor present more sensitive readouts to a defined DMMP concentration in spite of taking a longer time. However, when the amount is big enough, the sensing film is so thick that the DMMP molecules can only penetrate through the upper layer, and cannot reach to the bottom of the sensing film. Thus, the DMMP molecules will approach the equilibrium status of adsorption/desorption in the somewhat steady upper layer of the sensing film. Consequently, the effective surface adsorption area will no longer increase with the thickness of sensing films and the responses of the sensor to a given DMMP concentration tend to be stead too, shown as the plateau phase of the curve. The findings also illustrate that a sensing film with a thickness near to 125 μg/cm^2^ is most sensitive to 0.7 ppm of DMMP vapor. We recognized this thickness value as the optimal thickness of the ZnO-modified NW-structured MnO_2_ film coated on QCM under the predefined experimental conditions. We consequently thought that the gas sensors set at this working situation would have a maximized sensitivity to 0.7 ppm, therein designing the sensors with this thickness of sensing films for the measurement range containing 0.7 ppm and all other experiments in this study.

Anyway, how to find out the optimal thickness for designing QCM sensors with the best linearity and sensitivity to organic vapors is a challenge. As analyzed in the paragraphs above, despite many efforts, the gas diffusion and gas-surface interaction in sensing films are still far from being well understood for QCM metal oxide gas sensors. Further theoretical, empirical, or experimental results would be expected to understand how the gas concentration profile develops inside a thin film of metal oxide NWs after its exposure to a target gas, thus enabling us to design the sensing film more rationally.

### 3.4. Relationship between the sensitivity and concentration of DMMP vapor

**Figure 5.**
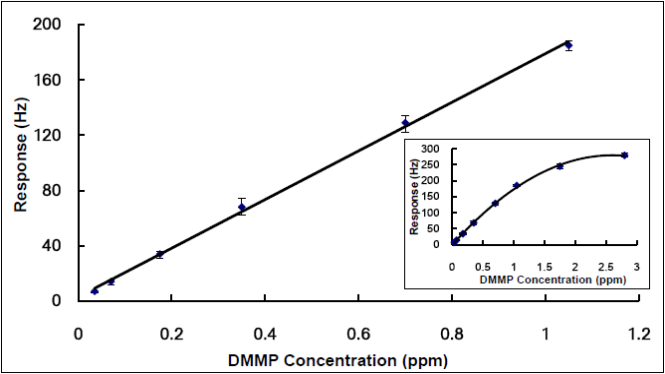
Linear plot of the reciprocal of a ZnO-modified nanowire MnO_2_ coated QCM sensor’s response against the concentrations of DMMP vapor purged in high-purity nitrogen at room temperature.

For each designed sensor, its sensitivity was assessed at various concentrations of DMMP vapors. In order to evaluate the repeatability, three or four consecutive measurements at each concentration were required. Thus, the response (R) to each given DMMP concentration (C) was repeatedly measured three or four times. The results over a range from 0.035 ppm to 2.8 ppm were shown in Figure 5, where the small plot indicated the QCM sensor based on ZnO-modified NW-structured MnO_2_ film began to illustrate discernible nonlinearity at a DMMP concentration of near 3 ppm.

In contrast, as shown in the large plot in Figure 5, the responses of the sensor were almost linearly proportional to the lower DMMP concentrations, ranging from 0.035 to 1.05 ppm. The regression equation could be expressed as R = 176.04 C + 3.01 with a correlation coefficient of 0.9987, where C is the concentration of DMMP vapors and R is the response or sensitivity of the sensor, respectively. This relationship plots the calibration curve of the ZnO-modified NW-structured MnO_2_ based QCM sensor. Accordingly, the limit of detection (calculated as three times the signal-to-noise ratio) could be estimated and given as 35 ppb.

This result represents a great improvement compared to those previously published by other groups. Brunol *et al.* reported their study to deal with the DMMP detection, using tin dioxide-based gas sensors. They used a DMMP vapor concentration level of around 200 ppm [6]. Ying *et al.* studied a PVDF coated QCM as the DMMP sensor [7]. The sensitivity was 3.19 Hz/ppm over the range from 5 to 60 ppm DMMP in N_2_ and the limit of detection was about 0.94 ppm. In comparison with their results, the sensor reported in this paper is highly sensitive, and thus suitable for low level DMMP detection.

Although the frequency change of QCM responded linearly to the amount of gas absorbed and illustrated a high sensitivity to DMMP vapors, this sensor demonstrated nonlinearity at a large concentration range of DMMP, which was different from the description based on the Sauerbrey equation [41]. This nonlinearity might be contributed to by the thickness effects of the sensing film because the QCM works on a balanced status between the sensitivity and linearity. In principle, the thicker thickness of ZnO-modified NW-structured MnO_2_ film possesses much more effective surface adsorption area to adsorb DMMP; this makes the QCM sensor much more sensitive. However, this thicker thickness also leads to the static working status of the QCM sensor being different from that of the theoretical QCM, thus producing slight nonlinear effects on the Sauerbrey equation. More importantly, as described in Section 3.3, a thicker sensing film also induces nonlinear DMMP adsorption. These facts indicate that the QCM DMMP sensor shows more observable nonlinearity. Thus the thickness of the sensing film is one of the key factors affecting not only the sensitivity, but also the linearity of a sensor.

### 3.5. Response of the sensor towards various organic vapors (selectivity)

**Figure 6.**
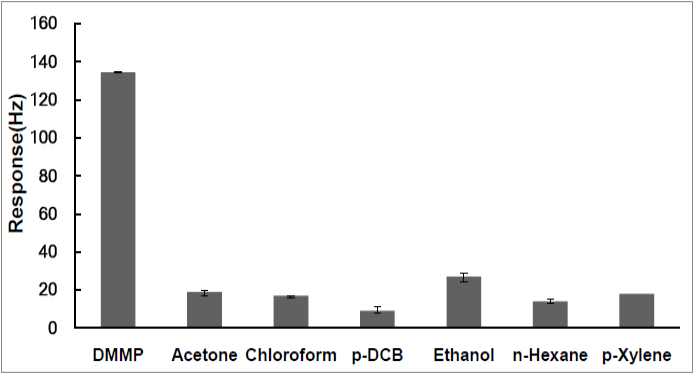
Comparison of the responses of the sensor to various organic vapors diluted to a predefined concentration of 0.7 ppm.

To investigate the selectivity, the QCM sensors were tested against several VOCs according to the instructions described above. The responses of the sensor exposed to a defined concentration of 0.7 ppm DMMP and potential interfering VOCs including acetone, chloroform, *p*-DCB, ethanol, *n*-hexane and *p*-xylene were measured. Each of these VOCs is usually used as solvents and may act as potential interferences. The amplitudes of the responses to the target gas––DMMP vapor as well as control vapors at same concentration of 0.7 ppm were shown as in Figure 6. As we can see from the Figure, the response to the target DMMP vapor was much larger than those to the acetone, chloroform, *p*-DCB, ethanol, *n*-hexane and *p*-xylene vapors. Therefore, these findings indicated that the developed ZnO-modified MnO_2_ NW-structured sensor possesses a very high selectivity to gaseous DMMP from VOCs including acetone, chloroform, *p*-DCB, ethanol, *n*-hexane and *p*-xylene.

### 3.6. Comparison of the sensitivity between the sensors coated with ZnO-modified MnO_2_ and pure MnO_2_

**Figure 7.**
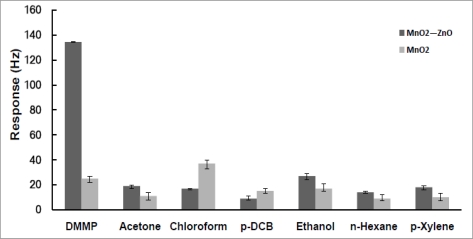
Comparison of the responses between the sensor based on pure MnO_2_ nanowire film and ZnO-modified MnO_2_ nanowire film to various organic vapor diluted to a predefined concentration of 0.7 ppm.

In order to assess the effect of the ZnO-modified MnO_2_ NW-structured film on DMMP sensing, a reference QCM sensor with a same structure but pure MnO_2_ NW film was fabricated for comparison. The responses of the reference QCM sensor to both the DMMP vapor and interfering VOCs including acetone, chloroform, *p*-DCB, ethanol, *n*-hexane and *p*-xylene at a same predefined concentration of 0.7 ppm were investigated according to the instructions described above. The response amplitudes of these paired QCM sensors were compared and are shown in Figure 7. The response of NW-structured pure MnO_2_-based QCM sensor to DMMP vapor was not higher than those to the potentially interfering VOCs; one example is that the sensitivity amplitude to the DMMP vapor was even slightly smaller than the one to chloroform. Clearly, these facts indicate that the QCM sensor based on NW-structured pure MnO_2_ did not present a usable selectivity to DMMP. In contrast, as shown in Figure 6, through the ZnO modification of MnO_2_ NWs, the sensitivity of the MnO_2_ NW film based QCM sensor to the DMMP vapor was greatly increased although that to the potentially interfering VOCs was almost unchanged. We thus contributed this improvement of the selectivity to DMMP to the ZnO modification on MnO_2_ NWs, or the formed composite nanostructures shown as in Figure 1.

Understanding the interaction of phosphonate esters with the surfaces of ZnO-modified MnO_2_ NWs at room temperature is a challenge; this problem is critical for the development of sensors to measure CWAs. However, very little is known about mechanism of DMMP specific binding to metal oxides although a few researchers have studied the details of DMMP absorbance. In this study, the MnO_2_ nanowires-based gas sensor responded to the VOCs very well in comparison with most gas sensors, but as displayed in Figure 6, presenting an average to low selectivity. In this study, the nanocrystalline ZnO was utilized to modulate the strong catalytic activity of MnO_2_, forming heterogeneous interfaces for VOCs testing. The heterogeneous interfaces formed possibly reduce the catalysis of MnO_2_ to some VOC vapors, but increase that to other VOC vapors. These distinctive properties of this developed composite nanostructured material were evaluated through the QCM gas sensing. They displayed as the sensitivity and selectivity of QCM sensor to DMMP on device level.

Interestingly, the experimental results indicated that the sensitivity and selectivity to the DMMP vapor was greatly increased whereas that to the VOC vapors was only slightly changed. We can thus contribute the improvement of the selectivity to the DMMP vapor to the modulation effects of the ZnO nanoparticles on the MnO_2_ NW-structured film. Of course, further study would be helpful to understand the detailed mechanisms at molecular level, and furthermore to improve the sensitivity and selectivity. Mitchell *et al.* have explored the uptake mechanisms of DMMP to the effective sorbent and reaction material TiO_2_ and considered that the DMMP molecule interacts through the electron-rich phosphoryl oxygen with surface-bound hydroxyl groups and with Lewis acid sites of the TiO_2_ [31]. Similar studies will be valuable to understand the mechanisms of DMMP effective absorption to the surface of the ZnO-modified MnO_2_ NWs at room temperature.

### 3.7. Long-term stability

**Figure 8.**
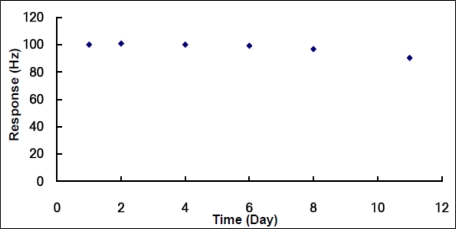
Responses of a nanowire ZnO_2_/MnO_2_ composite film based QCM sensor to a predefined concentration of 0.54 ppm DMMP in nitrogen gas during 10 days.

In order to assess the long-term stability, the response of the sensor to a predefined concentration of the DMMP in nitrogen gas, that is, 0.54 ppm, the average concentration of the linear range from 0.035 to 1.05 ppm, had been regularly examined for 10 days as the instructions described. After each testing, the sensor was immediately stored in a dry cabinet at room temperature. The response changes over ten-day period are shown in Figure 8. The results shown in Figure 8 indicated that the response was relative stable and remained above 90% of its original value after 10 days.

## 4. Conclusions

The preparation of highly active sensing film is believed to be a crucial step in obtaining sensitive and selective CWA sensors. For this purpose a composite nanostructured MnO_2_ material was produced in this study by the ZnO modification of MnO_2_ nanofibers. To evaluate its specific absorbing properties, both pure MnO_2_ and ZnO-modified MnO_2_ NW-structured films were prepared on QCMs for DMMP sensing. With the ZnO-modified device working at room temperature, a linear response to DMMP and a limit of detection of 35 ppb were obtained, even though the DMMP was diluted to a concentration lower than 1.05 ppm. The data presented in this study thus show that the ZnO modification induces the NW-structured MnO_2_ film effectively absorb DMMP vapor, thus improved significantly the sensitivity and selectivity of sensors. Therefore, this work provides valuable data with CWA simulant supporting the development of new nanostructured material as a sensitive film for the nerve agent and insecticide detection. The composite nanostructured DMMP-sensing film combined with the simple and low-cost QCM detection provides a promising configuration to develop practical chemical warfare gas sensor.
